# Off-Label Dextromethorphan/Bupropion for Emotional Lability Following Resection of an IDH-Mutant Astrocytoma: A Case Report

**DOI:** 10.3390/healthcare14203530

**Published:** 2026-10-20

**Authors:** Olivia Hill, Riley Imlay, Ankit Chalia, Sowjanya Kalluri, Venkata Sugnanam, Michael Ang-Rabanes, Raja Mogallapu

**Affiliations:** 1 Department of Behavioral Medicine, West Virginia University School of Medicine, Eastern Campus, Martinsburg, WV 25401, USA; o.hill967@gmail.com (O.H.); riley_imlay@brown.edu (R.I.); ankit.chalia@hsc.wvu.edu (A.C.); venkata.sugnanam@wvumedicine.org (V.S.); mangrabanes@hsc.wvu.edu (M.A.-R.); 2 Department of Hematology and Oncology, West Virginia University School of Medicine, Eastern Campus, Martinsburg, WV 25401, USA; sowjanya.kalluri@hsc.wvu.edu

**Keywords:** PBA, pseudobulbar affect, dextromethorphan, bupropion, combination of dextromethorphan, quinidine

## Abstract

Pseudobulbar affect (PBA) is characterized by recurrent, involuntary episodes of crying or laughing that may be disproportionate or incongruent with an individual’s underlying mood. Although dextromethorphan/quinidine is FDA-approved for PBA, clinical experience with dextromethorphan/bupropion for this indication remains limited. We describe a 42-year-old woman with a history of resected right frontal IDH-mutant grade 2 astrocytoma who developed recurrent episodes of involuntary crying associated with emotional lability and subjective executive difficulties. At initial behavioral-health evaluation, the Center for Neurologic Study–Lability Scale (CNS-LS) score was 21/35 and the Patient Health Questionnaire-9 (PHQ-9) score was 9. The phenomenology of the crying episodes, clinical examination, neurological history, imaging surveillance, and consideration of alternative explanations supported a working clinical diagnosis of PBA, although the CNS-LS was used as a symptom measure rather than a stand-alone diagnostic test. Treatment with dextromethorphan/quinidine was associated with an initial reduction in crying episodes and CNS-LS score, followed by recurrence of symptoms. Because further treatment modification was limited by insurance coverage, an off-label trial of dextromethorphan/bupropion was subsequently initiated. Six weeks later, the CNS-LS score had decreased to 10 and the PHQ-9 score to 4. Subjective executive difficulties also improved. A temporary interruption in dextromethorphan/bupropion was followed by recurrence of crying episodes, with improvement after treatment was resumed. This case describes an association between off-label dextromethorphan/bupropion exposure and improvement in emotional-lability symptoms in a patient with a complex neurological and psychiatric presentation. The findings are hypothesis-generating and cannot establish efficacy or causality because of the single-patient design, absence of repeated CNS-LS assessments at all visits, and concomitant medication changes. Controlled studies are needed to determine whether dextromethorphan/bupropion has a therapeutic role in PBA.

## 1. Introduction

Pseudobulbar affect (PBA) is a neurological syndrome characterized by involuntary and often disproportionate episodes of emotional expression, most commonly crying or laughing, that may be incongruent with an individual’s underlying emotional state or the surrounding social context. These episodes can result in substantial psychosocial distress, embarrassment, and functional impairment. Estimates of PBA prevalence vary depending on the underlying neurological population and diagnostic methodology, with approximately 1.8–7.1 million individuals estimated to be affected in the United States [1,2].

PBA has been described in association with multiple neurological disorders, including amyotrophic lateral sclerosis, multiple sclerosis, traumatic brain injury, stroke, dementia, and other neurological diseases [1,2,3,4]. The clinical presentation may overlap with psychiatric disorders, particularly major depressive disorder and other mood disorders, creating diagnostic challenges [2,3]. In contrast to sustained mood disturbance, PBA is characterized by recurrent, involuntary emotional expressions that may be disproportionate or incongruent with the individual’s underlying emotional state [1,3].

The pathophysiology of PBA is incompletely understood. Proposed mechanisms involve disruption of cortico-pontocerebellar and related cortico-limbic-subcortical networks involved in the regulation and modulation of emotional expression, with contributions from serotonergic and glutamatergic neurotransmission [3,4].

Frontal–subcortical circuitry is also relevant because these networks contribute to executive functioning, behavioral regulation, and emotional control [5].

Historically, antidepressants, including selective serotonin reuptake inhibitors and tricyclic antidepressants, have been used off label for PBA. Dextromethorphan/quinidine is an FDA-approved treatment for PBA and has demonstrated efficacy in randomized controlled trials [6,7].

Dextromethorphan has NMDA receptor antagonist and sigma-1 receptor agonist activity, while quinidine inhibits CYP2D6-mediated metabolism of dextromethorphan, thereby increasing its systemic exposure [7].

Dextromethorphan/bupropion is another combination containing dextromethorphan and the CYP2D6 inhibitor bupropion. Bupropion has been shown to substantially inhibit CYP2D6 activity [8]. The clinical development and pharmacological rationale for dextromethorphan/bupropion have primarily been described in the context of major depressive disorder [9]. Evidence specifically evaluating this combination for PBA remains limited.

We describe a case of recurrent involuntary crying and emotional lability in a patient with a history of right frontal IDH-mutant grade 2 astrocytoma who demonstrated symptomatic improvement following transition from dextromethorphan/quinidine to off-label dextromethorphan/bupropion.

This case therefore aims to describe the clinical course and treatment response while highlighting the diagnostic complexity, potential confounding factors, and safety considerations involved in the off-label use of dextromethorphan/bupropion for PBA-like emotional lability.

## 2. Case Presentation

A 42-year-old woman with a history of right frontal IDH-mutant grade 2 astrocytoma underwent surgical resection without subsequent chemotherapy or radiation therapy. She remained under neurological surveillance, with serial magnetic resonance imaging showing no evidence of tumor recurrence. Before the development of her neurological condition, she had no documented history of a primary psychiatric disorder. Following neurosurgical treatment, she developed recurrent episodes of involuntary crying associated with emotional lability. Initially, the episodes occurred approximately three to four times daily and lasted 20–30 min. Over time, their frequency increased to nearly hourly, with individual episodes lasting approximately 10–15 min. The crying was frequently unprovoked or disproportionate to the immediate circumstances and was described as difficult to control.

The episodes occurred without identifiable emotional or situational triggers and appeared incongruent with her underlying mood. For example, she reported that she could be having a routine conversation with her husband and suddenly begin crying without an identifiable precipitant. The episodes resulted in significant distress, embarrassment, and functional impairment. She also reported reduced motivation, feelings of helplessness and hopelessness, and subjective difficulties with attention, concentration, planning, and organization. She described these cognitive difficulties as resembling ADHD symptoms, However, no formal neuropsychological testing or standardized assessment of executive functioning was performed. Accordingly, these symptoms were considered subjective postoperative executive complaints rather than objectively established executive dysfunction. At the initial behavioral-health evaluation, the Patient Health Questionnaire-9 (PHQ-9) score was 9, indicating mild depressive symptom burden.

The PHQ-9 was interpreted as a measure of depressive symptom burden and was not used to establish a diagnosis of major depressive disorder, particularly given the absence of a prior history of major depressive disorder and the prominent phenomenology of the crying episodes. Her Center for Neurologic Study–Lability Scale (CNS-LS) score was 21/35 [10]. Although the CNS-LS is commonly used to assess emotional lability in neurological populations, it has been primarily validated in conditions such as amyotrophic lateral sclerosis and multiple sclerosis and has limitations as a stand-alone diagnostic instrument in patients with brain tumors. Therefore, the CNS-LS score was interpreted as supporting clinically significant emotional lability rather than independently confirming a diagnosis of pseudobulbar affect (PBA). The clinical diagnosis was based on the overall phenomenology and clinical context, including the recurrent and apparently involuntary nature of the crying episodes, their lack of identifiable situational or emotional triggers, their apparent incongruence with the patient’s underlying mood, her neurological history, clinical examination, and exclusion of alternative explanations. The differential diagnosis included major depressive disorder, postoperative frontal-lobe emotional dysregulation, tumor recurrence, medication-related behavioral or cognitive effects, seizure-related phenomena, pseudobulbar palsy, and PBA. Screening did not identify symptoms suggestive of bipolar disorder, psychosis, or substance-related causes.

The patient had no history of seizures before or after tumor resection. Levetiracetam was initially prescribed following surgery for seizure prophylaxis despite the absence of documented seizures and was subsequently discontinued. She remained on other medications used in the management of postoperative headaches and associated symptoms. Neurological and mental-status examinations were within normal limits. There was no evidence of dysarthria, dysphagia, facial or tongue weakness, or other corticobulbar findings, making pseudobulbar palsy less likely [11].

### 2.1. Initial Pharmacologic Treatment

The patient was initially treated with dextromethorphan/quinidine, the FDA-approved pharmacologic treatment for pseudobulbar affect (PBA). The labeled regimen consists of one 20-mg/10-mg capsule once daily for the first 7 days, followed by one capsule every 12 h thereafter. In this case, the patient received dextromethorphan/quinidine 20 mg/10 mg once daily for the first 3 days. Because she reported a partial response and desired more rapid symptom improvement while continuing to work, a shared clinical decision was made to increase the medication to twice-daily dosing before completion of the standard 7-day once-daily initiation period. This represented a deviation from the labeled initiation regimen and reflects the treatment received by the patient; it should not be interpreted as a recommended alternative dosing strategy. Trazodone at 25 mg nightly as needed was prescribed for sleep difficulties. The patient’s neurologic medication regimen also changed during this period. Levetiracetam had initially been prescribed postoperatively for seizure prophylaxis despite no documented history of seizures. An EEG performed after surgery was negative for epileptiform activity.

At the 3-week follow-up, the patient reported fewer crying episodes and improved emotional regulation. Her CNS-LS score decreased from 21/35 at baseline to 14/35. Topiramate was subsequently initiated by neurology for headache management while verapamil was continued along with Levetiracetam.

Approximately 4 weeks later, however, the crying episodes recurred; the CNS-LS was not repeated at that visit. Further modification of dextromethorphan/quinidine therapy was limited by insurance coverage. Valproic acid was subsequently introduced, and topiramate and Levetiracetam were discontinued in the context of ongoing postoperative headaches and post prophylaxis seizures.

Because neurological medications were initiated, discontinued, or adjusted during the overall treatment period, their potential contributions to the patient’s emotional and cognitive symptoms cannot be completely separated from the effects temporally associated with dextromethorphan/quinidine. Accordingly, the observed improvement during this phase cannot be attributed with certainty to dextromethorphan/quinidine alone.

### 2.2. Transition to Dextromethorphan/Bupropion

Because of persistent or recurrent crying episodes and limitations in further adjustment of dextromethorphan/quinidine therapy, the patient was transitioned to dextromethorphan/bupropion. Unlike dextromethorphan/quinidine, dextromethorphan/bupropion is not FDA-approved for the treatment of PBA. Its FDA-approved indication is major depressive disorder. The pharmacologic rationale for this off-label approach was based in part on bupropion’s potent inhibition of cytochrome P450 2D6 (CYP2D6), which increases dextromethorphan exposure, together with bupropion’s norepinephrine–dopamine reuptake inhibition. The antidepressant activity of bupropion was potentially relevant to the patient’s depressive symptom burden. However, because bupropion has independent antidepressant activity, the relative contributions of dextromethorphan and bupropion to the observed clinical response cannot be isolated. The patient was prescribed the fixed-dose combination of dextromethorphan at 45 mg/bupropion at 105 mg daily for the first week, with the dose subsequently increased to twice daily as tolerated.

Approximately 6 weeks after initiation of dextromethorphan/bupropion, the patient reported a substantial reduction in the frequency and severity of crying episodes. At this assessment, her CNS-LS score had decreased from 21/35 at baseline to 10/35, and her PHQ-9 score had decreased from 9 to 4. CNS-LS and PHQ-9 assessments were not repeated during the third session because the patient was markedly tearful and emotionally distressed. At the subsequent session, approximately 2 weeks after initiation of dextromethorphan/bupropion, the clinical team deferred repeat assessment to allow additional time for treatment response. The standardized measures were subsequently repeated at approximately 6 weeks, demonstrating improvement in both emotional lability and depressive symptom burden.

These changes represented improvement in measured emotional-lability and depressive symptom burden, respectively, but did not independently establish remission of PBA or major depressive disorder. The patient also reported subjective improvement in attention, concentration, planning, and organization. Because these symptoms were not evaluated using formal neuropsychological testing or a standardized measure of executive functioning, these findings are presented as subjective clinical observations rather than objective evidence of improved executive functioning.

### 2.3. Treatment Interruption and Follow-Up

During subsequent follow-up, the patient experienced a temporary interruption in dextromethorphan/bupropion treatment because of difficulty obtaining a medication refill. During the interruption, her crying episodes recurred. Following resumption of dextromethorphan/bupropion, she reported prompt improvement in the crying episodes. The temporal association among medication interruption, recurrence of symptoms, and improvement following medication resumption provides additional observational support for a relationship between medication exposure and symptom control. However, this temporal association does not establish causality (Table 1).

## 3. Diagnostic Considerations

### 3.1. Tumor Recurrence

The symptoms of depression and executive dysfunction in this patient raised concerns about potential tumor recurrence. Her history of a low-grade IDH-mutant astrocytoma in the right frontal lobe, resected without subsequent chemotherapy or radiotherapy, necessitated close monitoring for recurrence. Several factors complicated diagnostic interpretation. First, the patient’s history of a right frontal astrocytoma and neurosurgical treatment provided a structural neurological substrate that could plausibly contribute to emotional and executive dysfunction. Frontal–subcortical circuits are involved in executive and behavioral regulation [5], while gliomas and their treatment may affect cognition and capacity [12].

**Figure 1:**
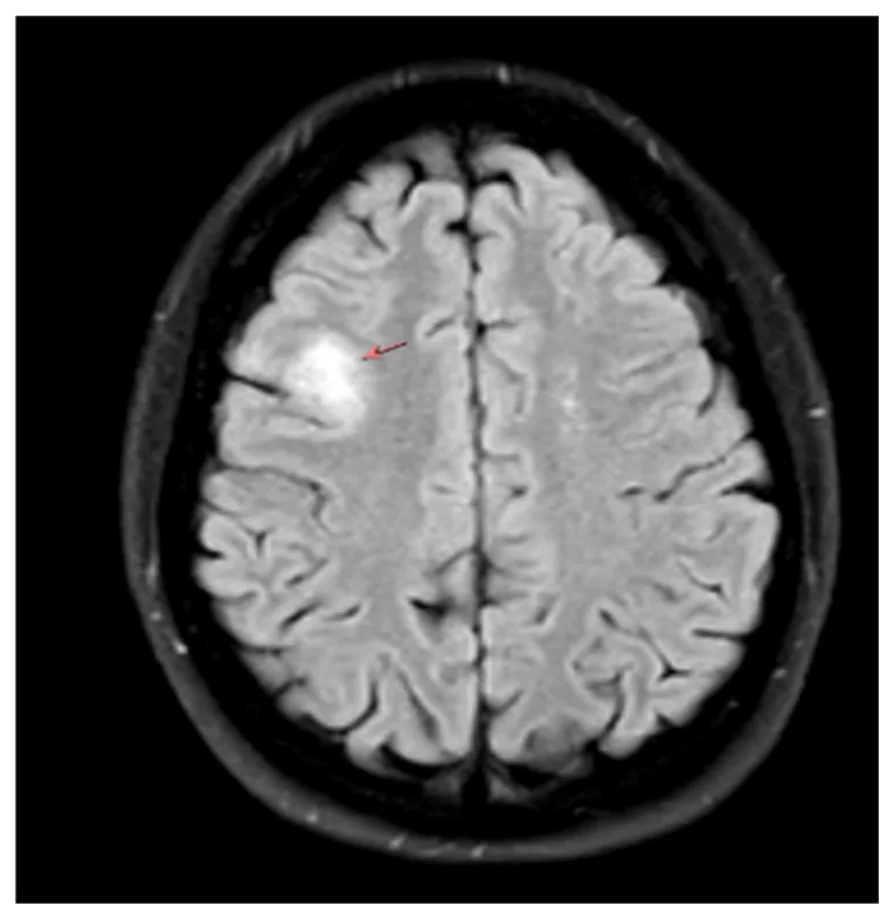
Axial brain MRI FLAIR image: Expansile, non-enhancing mass in right frontal cortex.

**Figure 2:**
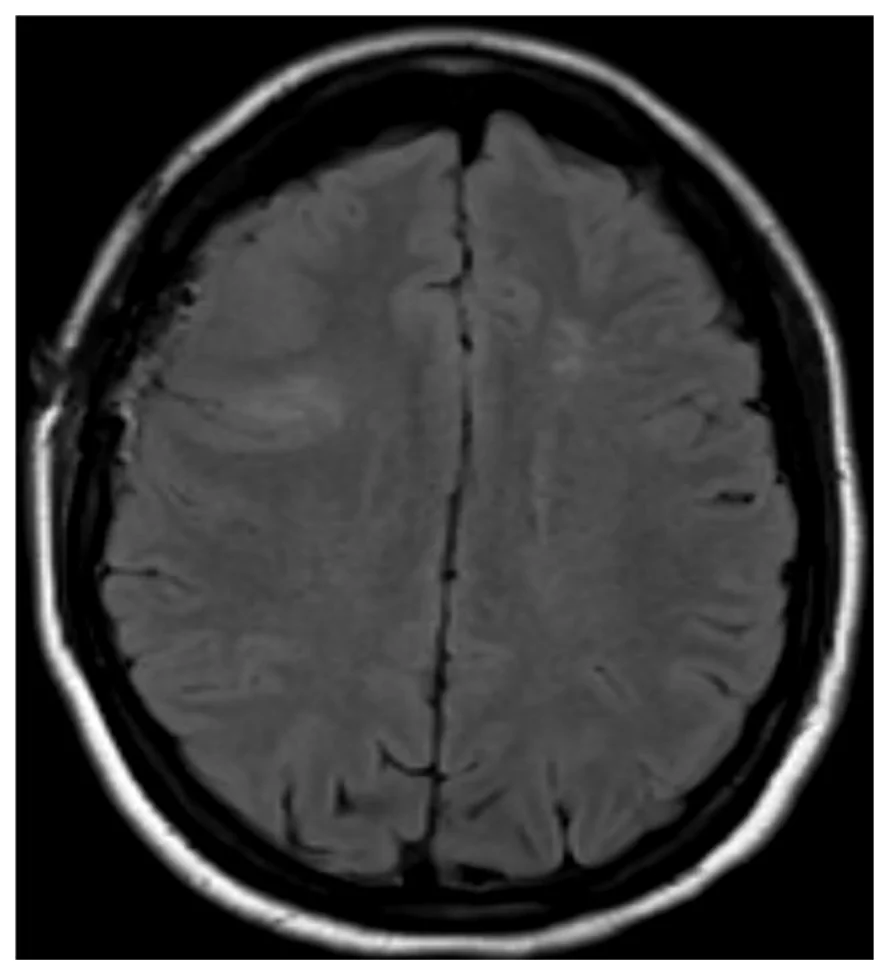
Axial brain MRI FLAIR image: Postoperative phase change from right frontal low-grade glioma.

Routine MRI scans by her neurology and oncology teams have shown no evidence of recurrence, consistent with the favorable prognosis (Figure 1 and Figure 2).

### 3.2. Depression

Differentiating pseudobulbar affect (PBA) from depression is critical, given the overlap in symptoms such as frequent crying episodes. In this case, the Patient Health Questionnaire-9 (PHQ-9) indicated mild depression, but the crying episodes lacked identifiable triggers and were out of proportion to her overall mood, strongly suggesting PBA. Depression is a common comorbidity in patients with brain tumors, particularly when frontal lobe involvement impacts mood regulation [12]. However, her emotional episodes were brief and recurrent, distinguishing them from the more prolonged affective episodes typical of depression [13] (Table 2).

### 3.3. Medication-Related Behavioral Effects

Levetiracetam and topiramate were considered potential contributors to behavioral or affective changes, and subsequent treatment with valproic acid represented another potential confounder. Because these medications were changed during the period of observation, their effects cannot be completely separated from the effects of dextromethorphan/bupropion [14,15].

### 3.4. Seizure Disorders

Frontal or limbic seizures can present with behavioral or emotional manifestations, including stereotyped episodes of crying (dacrystic seizures) [16]. The patient had no documented history of seizures, although levetiracetam had been prescribed postoperatively for seizure prophylaxis. A postoperative EEG was negative for epileptiform activity. The absence of documented seizures, altered awareness, or other clear ictal features made a seizure-related explanation less likely clinically. However, a negative routine EEG does not definitively exclude epilepsy, particularly in frontal lobe epilepsy; therefore, seizure-related phenomena remained a consideration in the differential diagnosis.

### 3.5. Pseudobulbar Palsy

Pseudobulbar palsy, a disorder involving the upper motor neurons, can present with emotional lability, dysarthria, dysphagia, and facial or tongue weakness. In this case, the absence of these motor symptoms makes pseudobulbar palsy unlikely [11].

### 3.6. Postoperative Frontal-Lobe Emotional Dysregulation

Given the patient’s history of a right frontal astrocytoma and surgical resection, postoperative frontal-lobe dysfunction was considered an important alternative explanation. The frontal lobes play an important role in emotional regulation, behavioral inhibition, attention, and executive functioning, and disruption of frontal network systems can result in abnormalities of affect and behavior [5]. Therefore, the possibility that the patient’s symptoms represented postoperative frontal-lobe emotional dysregulation rather than isolated PBA could not be completely excluded.

### 3.7. Pseudobulbar Affect

Overall, the clinical presentation was considered consistent with PBA as a clinical working diagnosis, while recognizing potential contributions from postoperative frontal-lobe dysfunction, depressive symptoms, medication effects, and other neurological factors. The working diagnosis was based primarily on the episodic phenomenology of involuntary crying, its relationship to the patient’s baseline mood and context, and the neurological history. The baseline CNS-LS score of 21 supported clinically significant emotional lability but was not considered diagnostic of PBA. Similarly, the subsequent reduction in CNS-LS score was interpreted as evidence of symptomatic improvement rather than confirmation of the diagnosis.

## 4. Discussion

This case illustrates the diagnostic and therapeutic complexity of recurrent emotional lability in a patient with structural neurological disease. The patient’s recurrent, brief, involuntary crying episodes were disproportionate to the surrounding circumstances and occurred in the context of a right frontal structural brain lesion and prior neurosurgical treatment. These features were clinically compatible with PBA, while several alternative explanations required consideration. PBA is a clinical syndrome diagnosed on the basis of characteristic symptoms and clinical assessment, rather than a condition established by a single laboratory or imaging test. The CNS-LS can be useful for quantifying affective lability and monitoring symptom change but should not be interpreted as a stand-alone diagnostic instrument [10].

In this case, the baseline CNS-LS score of 21/35 and subsequent reductions to 14 following dextromethorphan/quinidine and to 10 following dextromethorphan/bupropion provided quantitative measures of symptom change. The patient’s PHQ-9 score of 9 indicated mild depressive symptoms at baseline. However, the phenomenology of her crying episodes differed from a sustained depressive affective state. The episodes were recurrent, relatively discrete, involuntary, and often incongruent with the immediate emotional context. These characteristics supported PBA as a clinical working diagnosis while recognizing that depressive symptoms and PBA may coexist [2,3].

The history of right frontal astrocytoma was particularly relevant to the differential diagnosis. Frontal–subcortical networks participate in executive functioning, behavioral regulation, and emotional control [5]. Gliomas and their treatment can also affect cognition through multiple mechanisms, making postoperative frontal-lobe dysfunction an important alternative explanation for the patient’s subjective executive complaints [14]. Tumor recurrence was considered but was not supported by surveillance neuroimaging. Seizure-related phenomena were also considered because of the underlying structural brain lesion. The patient had no established seizure history, and EEG did not demonstrate epileptiform activity. The absence of epileptiform activity reduced support for a seizure-related explanation but did not completely exclude epilepsy. The absence of dysarthria, dysphagia, facial weakness, and tongue weakness made pseudobulbar palsy less likely [11].

The pharmacological response provided additional clinical information but should not be interpreted as diagnostic proof. The patient’s initial response to dextromethorphan/quinidine was followed by recurrence of symptoms, while subsequent treatment with dextromethorphan/bupropion was associated with a greater reduction in CNS-LS score. The recurrence of symptoms during a temporary treatment interruption and improvement after treatment resumption further strengthened the temporal association. Nevertheless, the uncontrolled nature of the observation, concomitant medications, and absence of objective neuropsychological testing prevent definitive attribution of the clinical changes to dextromethorphan/bupropion. Dextromethorphan has NMDA receptor antagonist and sigma-1 receptor agonist activity. Quinidine inhibits CYP2D6-mediated metabolism of dextromethorphan, thereby increasing dextromethorphan exposure [7]. Bupropion is also a clinically significant CYP2D6 inhibitor [8].

The dextromethorphan/bupropion combination has been studied primarily as an antidepressant treatment, with proposed pharmacological effects involving NMDA receptor antagonism, sigma-1 receptor agonism, and monoaminergic mechanisms [9]. However, these data do not establish efficacy for PBA. Controlled studies have established the efficacy of dextromethorphan/quinidine for PBA [6,7]. By contrast, evidence specifically evaluating dextromethorphan/bupropion for PBA remains limited. The available literature on dextromethorphan/bupropion has primarily addressed major depressive disorder rather than PBA [9]. Therefore, the improvement observed in this patient should be considered hypothesis-generating rather than evidence of established efficacy for PBA.

The patient’s subjective improvement in attention, concentration, planning, and organization is also noteworthy but should be interpreted cautiously. No formal neuropsychological testing was performed, and therefore these observations cannot establish objective cognitive improvement. Additionally, prior exposure to levetiracetam and topiramate may have influenced subjective cognitive or behavioral functioning [14]. The patient’s trial of valproic acid is also noteworthy. Although valproic acid is not an established evidence-based treatment for PBA, Johnson and Nichols reported a patient with multiple sclerosis whose PBA symptoms resolved following treatment with valproic acid [15]. In that report, symptoms subsequently recurred after transition to dextromethorphan/quinidine [15]. In contrast, valproic acid did not produce a clear improvement in the crying episodes in our patient. These differing observations emphasize the limited and heterogeneous nature of the evidence regarding valproic acid and PBA and do not support a definitive conclusion regarding its efficacy. The patient’s improvement in PHQ-9 score from 9 to 4 is also relevant. Because dextromethorphan/bupropion has established antidepressant effects [9], improvement in depressive symptoms could have contributed to the overall clinical improvement. However, the episodic and context-incongruent nature of the crying, together with the reduction in CNS-LS score, suggests that the change in affective lability cannot necessarily be attributed solely to improvement in depression. The temporary interruption and subsequent recurrence of symptoms provide an additional temporal observation. However, because this was an uncontrolled single-patient observation, the treatment interruption and subsequent improvement cannot establish a causal relationship.

The case also highlights the potential complexity of pharmacological management in patients with structural neurological disease. Concomitant antiseizure medications, headache treatments, depressive symptoms, and the underlying neurological lesion may all influence emotional and cognitive functioning. Accordingly, treatment response should be interpreted within the broader neurological and psychiatric context rather than as evidence of a specific mechanism.

Safety is another important consideration when using dextromethorphan-containing medications. Dextromethorphan can produce dissociative or hallucinogenic effects at substantially higher-than-therapeutic doses and has been associated with misuse, particularly when obtained from over-the-counter formulations [17]. In this case, the negative urine drug screen, absence of a documented history of substance misuse or seizures, normal laboratory values and ECG, and negative EMG findings provided additional clinical context when assessing potential medication-related risks and the overall clinical picture. Because trazodone was used concurrently, the potential for serotonergic toxicity with dextromethorphan was also considered and monitored. No clinical evidence of serotonin syndrome was observed during follow-up.

Several limitations should be acknowledged. First, this is a single case and therefore cannot establish efficacy or generalizability. Second, formal neuropsychological testing was not performed, limiting interpretation of the reported cognitive improvement. Third, the patient received multiple medications during the observation period, creating potential confounding. Fourth, the CNS-LS was not consistently repeated at every stage of treatment.

Finally, the diagnosis of PBA remained a clinical working diagnosis rather than one confirmed by a disease-specific biomarker. Despite these limitations, the temporal association between treatment with dextromethorphan/bupropion and improvement in both the frequency of crying episodes and CNS-LS score provides a clinically relevant observation. The case may help generate hypotheses regarding the potential role of dextromethorphan/bupropion in emotional lability associated with structural neurological disease.

## 5. Conclusions

This case describes improvement in recurrent emotional-lability symptoms following a transition from dextromethorphan/quinidine to off-label dextromethorphan/bupropion in a patient with a history of right frontal IDH-mutant astrocytoma. Although the clinical presentation was compatible with PBA, the diagnosis remained a clinical working diagnosis given overlapping postoperative, depressive, seizure-related, and medication-related considerations. The improvement in CNS-LS and PHQ-9 scores, along with symptom recurrence during a brief treatment interruption and improvement after treatment resumption, suggests a temporal association with dextromethorphan/bupropion treatment. However, causality cannot be established because of concomitant medications, limited repeated assessments, and the independent antidepressant effects of bupropion. Subjective improvements in attention and executive functioning were not objectively assessed. This single case does not establish dextromethorphan/bupropion as a treatment for PBA but highlights a potential area for further clinical investigation, particularly in patients with emotional lability and coexisting depressive symptoms following neurological injury. Further research, including randomized controlled trials, is needed to determine the potential role of dextromethorphan/bupropion in PBA, particularly in patients with structural neurological diseases.

## Figures and Tables

**Table 1:** Visit Progression.

Clinical Time Point	Medications at/Preceding the Visit	CNS LS	PHQ-9	Symptom Progression
1. Baseline	Levetiracetam 500 mg BID; acetaminophen; verapamil, dextromethorphan/quinidine 20 mg/10 mg once daily for three days followed by twice daily	21	9	Frequent, sudden, and difficult-to-control crying episodes without identifiable emotional or situational triggers, accompanied by irritability. Episodes initially occurred approximately 3–4 times daily and subsequently increased to nearly hourly. Episodes initially lasted approximately 20–30 min and later approximately 10–15 min.
2. 3-week follow-up	Levetiracetam; acetaminophen; verapamil 80 mg BID; topiramate 50 mg BID; dextromethorphan/quinidine 20 mg/10 mg twice daily; trazodone 25 mg nightly as needed	14	6	Crying episodes and irritability **significantly decreased,** with improved emotional regulation.
**3.** Approximately 4 weeks later	Valproic acid 250 mg BID; acetaminophen; verapamil; dextromethorphan/quinidine20 mg/10 mg twice daily	Not assessed	Not assessed	Crying episodes **recurred**. Further optimization of dextromethorphan/quinidine was limited by insurance coverage.
4. Subsequent follow-up 2 weeks later after transition to dextromethorphan/bupropion	Valproic acid; acetaminophen; verapamil; dextromethorphan/bupropion 45 mg/105 mg twice daily	Not assessed	Not assessed	Crying episodes decreased following the medication adjustment.
5. Subsequent follow-up 6 weeks later after transition to dextromethorphan/bupropion	Valproic acid; acetaminophen; verapamil; trazodone 25 mg nightly as needed; dextromthorphan/bupropion 45 mg/105 mg twice daily	10	4	Marked improvement in crying episodes and emotional lability. The PHQ-9 decreased from 9 at baseline to 4. The patient also reported subjective improvement in attention, concentration, planning, and organization.

**Table 2:** Differentiating between depression and PBA.

Aspect of:	Depression	PBA
**Duration of emotional distress**	Weeks to years	Seconds to minutes, recurring
**Cause of emotional episodes**	Mood disorder/related to context	Unknown cause, out of context
**Typical mood**	Low	Euthymic (outside of episodes)
**Behavior**	Has coexisting alterations in appetite, sleep, energy levels, psychomotor slowing, lack of interest in usual activities, decreased concentration, feelings of hopelessness, and/or suicidal ideation	Regular outside of episodes
**FDA-approved treatments**	SSRIs, SNRIs, TCAs, MAOIs, NMDA antagonists, Bupropion, Mirtazapine, Trazodone	Combination of dextromethorphan and quinidine 20 mg/10 mg

## Data Availability

The data presented in this study are available on request from the corresponding author because privacy restrictions.
